# GASZ self-interaction clusters mitochondria into the intermitochondrial cement for proper germ cell development

**DOI:** 10.1093/pnasnexus/pgad480

**Published:** 2024-01-02

**Authors:** Junru Miao, Chuanyun Wang, Wei Chen, Yongsheng Wang, Shalin Kakasani, Yuan Wang

**Affiliations:** Department of Animal Sciences, College of Agriculture and Natural Resources, Michigan State University, East Lansing, MI 48824, USA; Shanghai Key Laboratory of Regulatory Biology, Institute of Biomedical Sciences and School of Life Sciences, East China Normal University, Shanghai, 200241, China; Department of Animal Sciences, College of Agriculture and Natural Resources, Michigan State University, East Lansing, MI 48824, USA; Shanghai Key Laboratory of Regulatory Biology, Institute of Biomedical Sciences and School of Life Sciences, East China Normal University, Shanghai, 200241, China; Department of Animal Sciences, College of Agriculture and Natural Resources, Michigan State University, East Lansing, MI 48824, USA; Shanghai Key Laboratory of Regulatory Biology, Institute of Biomedical Sciences and School of Life Sciences, East China Normal University, Shanghai, 200241, China

**Keywords:** GASZ, piRNA, mitochondria, intermitochondrial cement

## Abstract

Mitochondrial features and activities vary in a cell type- and developmental stage-dependent manner to critically impact cell function and lineage development. Particularly in male germ cells, mitochondria are uniquely clustered into intermitochondrial cement (IMC), an electron-dense granule in the cytoplasm to support proper spermatogenesis. But it remains puzzling how mitochondria assemble into such a stable structure as IMC without limiting membrane during development. Here, we showed that GASZ (germ cell-specific, ankyrin repeat, SAM and basic leucine zipper domain containing protein), a mitochondrion-localized germ cell-specific protein, self-interacted with each other to cluster mitochondria and maintain protein stability for IMC assembling. When the self-interaction of GASZ was disrupted by either deleting its critical interaction motif or using a blocking peptide, the IMC structure was destabilized, which in turn led to impaired spermatogenesis. Notably, the blocked spermatogenesis was reversible once GASZ self-interaction was recovered. Our findings thus reveal a critical mechanism by which mitochondrion-based granules are properly assembled to support germ cell development while providing an alternative strategy for developing nonhormonal male contraceptives by targeting IMC protein interactions.

**Significance Statement**

Germ cell development requires properly assembled intermitochondrial cement (IMC), the failure of which impairs male fertility. This study reveals a critical mechanism by which mitochondria are correctly assembled into IMC via GASZ self-interaction to support proper germ cell development, thus informing mechanistic causes of male infertility due to dysfunctional protein interactions for targeted therapies. Study findings may also provide a new target for developing nonhormonal reversible male contraceptives.

## Introduction

Properly developed germ cell lineage ensures the faithful passage of genetic information through generations. In mammals, the initial germ cell population, known as primordial germ cells, arises during the embryonic stage and develops into prospermatogonia in male gonads (1, 2). After birth, in spermatogenesis, a small fraction of prospermatogonia become spermatogonial stem cells to maintain a pool of undifferentiated progenitor spermatogonia (2). These undifferentiated spermatogonia further go through stages of differentiation, two consecutive meiosis, before maturing into spermatids (3). In mice, primary spermatocytes from differentiating type B spermatogonia undergo long meiotic prophase I, passing through the stages of leptotene, zygotene, pachytene, diplotene, and diakinesis, followed by two chromosome divisions to finally produce haploid spermatids (2, 3). Blocking any of these steps in this process will disrupt germ cell development and cause male infertility.

Mitochondrial features, including their morphology, architecture, numbers, subcellular localization, and interaction with other organelles, vary in a cell type and developmental stage-dependent manner to critically affect the mitochondrial functional state (4). In male germ cells, nuage, the amorphous electron-dense granule with no confining membrane has been identified in the cytoplasm using transmission electron microscopy (TEM) (5–9). The intermitochondrial cement (IMC) is a specific type of nuage, where clustered mitochondria with ribonucleoproteins as the “cementing materials” are uniquely present in prospermatogonia, postnatal spermatogonia, and pachytene spermatocytes (5, 6). IMC has been known to participate in the biogenesis of PIWI-interacting RNAs (piRNAs), a class of 24–32 nt noncoding small RNAs identified in germ cells from diverse species (10–15). The piRNAs, especially prepachytene piRNAs, maintain germline DNA integrity by repressing the expression of transposable elements, a function essential for germ cell development (10–15). Disrupting IMC formation or removing piRNA processing proteins from IMC often blocks spermatogenesis and impairs male fertility (16–24). Recently, it has also been reported that IMC-localized proteins directly regulate mitochondrial activities and metabolism or indirectly via interacting with other mitochondrial proteins, which in turn impact spermatogenesis (23, 25, 26).

It has been long known that the mitochondrial clustering at IMC is a unique feature of male germ cells (5, 7). However, it remains puzzling how mitochondria cluster into such a stable structure at IMC without confining membrane from embryonic germ cells to pachytene spermatocytes during development. To date, only a few germ cell-specific proteins are known to be localized directly at mitochondria. Of these, GASZ (also called ASZ1) is found at the mitochondrial outer membrane *via* a C-terminal mitochondrial localization signal (MLS) (23, 27). GASZ is a germ cell-specific protein with four Ankyrin repeats (ANK), a sterile alpha motif (SAM), and a putative basic leucine zipper domain (bZIP) (28). Deleting MLS from GASZ disrupts IMC formation, reduces piRNA biogenesis, and leads to male infertility, supporting that proper mitochondrial localization of GASZ is essential for IMC construction and germ cell development (22, 23). GASZ proteins are readily detectable at embryonic day 13.5 (29). It is, to date, the only known germ cell-specific mitochondrial protein that affects IMC formation in embryonic germ cells and postnatal spermatogonia. In this study, we demonstrated that GASZ formed dimers to enable mitochondrial clustering for IMC assembling. When GASZ dimerization was disrupted, IMC was destabilized, accompanied by aberrant piRNA biogenesis and blocked spermatid development. Notably, we also found that this blocked germ cell development could be reversed when GASZ self-interaction was resumed, providing an alternative strategy for developing nonhormonal reversible male contraceptives by targeting IMC protein interactions.

## Results

### GASZ proteins self-interact to form dimers

**Fig. 1.**
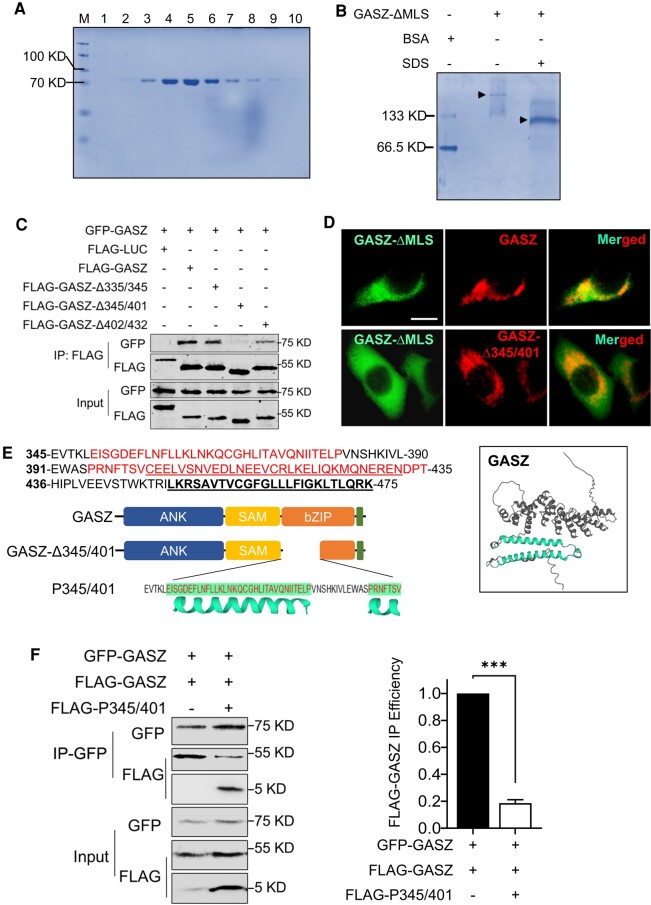
GASZ proteins self-interact to form dimers in the absence of other germ cell-specific proteins. A) Ni-NTA resin-purified protein fractions were collected from gel filtration and subjected to SDS-PAGE. Total proteins were visualized with Coomassie blue staining. M, protein ladder. B) HIS-MBP-tagged GASZ-ΔMLS collected from fraction 5 in (A) were subjected to native PAGE with or without 2% SDS in the loading buffer, followed by Coomassie blue staining. Bovine serum albumin was used as a marker to indicate the molecular weight of protein bands. C) GFP-GASZ fusion proteins were co-expressed with FLAG-tagged full-length GASZ (FLAG-GASZ) or GASZ truncated mutants in 293 T cells. Cell lysates were IP with FLAG affinity beads for WB analyses. Antibodies used for WB were shown on the left. FLAG-LUC, FLAG-luciferase. D) GFP-GASZ-ΔMLS fusion proteins were co-expressed in HeLa cells with FLAG-GASZ or FLAG-GASZ-Δ345/401. Protein subcellular localization was visualized by IF using antibodies against GFP and FLAG (red). Representative cell images were shown. Scale bar: 25 μm. E) Protein sequences that encode the bZIP (α-helices in red fonts) and MLS of GASZ. Underlined red fonts: previously reported bZIP sequences. Underlined black fonts: MLS. A graph shown below the protein sequences to illustrate the functional domains of full-length GASZ protein, GASZ-Δ345/401, and the peptide (P345/401) corresponding to GASZ self-interaction domain. The GASZ protein structure predicted by AlphaFold 2 was shown on the right, with the basic leucine zipper in green. F) GFP- and FLAG-tagged full-length GASZ were co-expressed in 293 T cells in the absence (−) or presence (+) of a FLAG-P345–401. The cell lysate was IP using a GFP antibody for WB analyses. Left panel: target proteins were visualized and determined based on their molecular weights in WB using antibodies against FLAG or GFP. Right panel: relative protein levels of FLAG-GASZ that were co-IP with GFP-GASZ by a GFP antibody were determined via grey-scale intensity of protein bands in Western Blots. Data were presented as mean ± SEM from three independent experiments; ***P < 0.001.

Our previous study demonstrated that the mitochondrion-localized GASZ proteins interacted with each other to promote mitochondrial aggregation in somatic cells (23). Because GASZ is a known IMC protein, we hypothesize that GASZ proteins self-interact and then cluster mitochondria to enable IMC assembling for piRNA biogenesis. To confirm that GASZ indeed self-interacts in the absence of other germ cell-specific proteins, we purified GASZ proteins from bacteria (fraction 5 in Figs. 1A and S1A and B). To improve its solubility and stability, we deleted the transmembrane MLS from the GASZ protein and then fused GASZ-ΔMLS with a HIS (polyhistidine)-MBP, the commonly used tags that enhance protein solubility (30). HIS-MBP-GASZ-ΔMLS was the only band seen from samples collected *via* Ni-NTA (Ni^2+^-charged agarose) resin purification and gel filtration (Fig. 1A), suggesting no contamination from other bacterial proteins. We ran the purified proteins through native polyacrylamide gel electrophoresis (PAGE) and found that the molecular weight of the main protein band (∼160 KD) without any denaturing reagent was about twice that of GASZ monomer (∼80 KD) denatured by SDS (sodium dodecylsulfate) (Fig. 1B), proving that GASZ formed dimers via direct self-interaction.

We further determined the critical regions of GASZ proteins that mediated their self-interaction. As shown by co-immunoprecipitation (Co-IP) assays, mutant GASZ proteins without amino acid (aa) 345 to 401 (GASZ-Δ345/451) could not interact with the full-length GASZ (Fig. 1C). In addition, cytoplasmic GASZ mutant without its MLS (GASZ-ΔMLS) could be readily brought to mitochondria by the full-length mitochondrion-localized GASZ *via* self-interaction (Figs. 1D and S1C). By contrast, when GASZ-ΔMLS proteins were co-expressed with mitochondrion-localized GASZ-Δ345/401, they were no longer recruited to mitochondria, suggesting aa 345–401 region of GASZ is key for its self-interaction. These results were further confirmed by a bimolecular fluorescence complementation assay, in which YFP (yellow fluorescent protein) was split into half and emitted fluorescence only when these two halves were in proximity to each other [36]. In this assay, we included a cytoplasmic GASZ mutant (GASZ-ΔMLS) to avoid artificial fluorescence that could be produced by the full-length GASZ proteins localized in proximity at mitochondria. Indeed, fluorescence was barely observed when NYFP (N-terminal half of YFP)-GASZ-ΔMLS and CYFP (C-terminal half of YFP) were cotransfected into cells (Fig. S2A). By contrast, when the N- and C-terminal halves of YFP were fused to full-length GASZ and GASZ-ΔMLS, respectively, and co-expressed, substantially more cells with brighter fluorescence were observed (Fig. S2A). Such a phenomenon could not be observed when GASZ interaction mutant (CYFP-GASZ-Δ345/401) was co-expressed with NYFP-GASZ-ΔMLS (Fig. S2A). The GASZ self-interaction region (aa 345–401) is located between the previously predicted SAM and bZIP domains (28). However, when using AlphaFold 2 in Google Colab (31) to deduce 3D protein structure of GASZ proteins, we found that the previously predicted bZIP sequences (aa 402–432) only contained part of one α-helix. The newly identified self-interaction region (aa 345–401) formed another α-helix and the linker region between the two α-helices (Fig. 1E, left panel). These two parallel α-helices comprise the entire basic leucine zipper motif of GASZ (Fig. 1E, right panel).

It is possible that the deletion of aa 345–401 in mutant GASZ changes its protein structure and thus abolishes its ability to self-interact. To exclude this possibility, we expressed a FLAG-tagged peptide only containing aa 345 to 401 (FLAG-P345/401), the self-interaction domain of GASZ. We found this peptide was readily co-IP with the full-length GFP (green fluorescent protein)-GASZ by an antibody against GFP (Fig. 1F). Interestingly, we also observed significantly reduced interaction between GFP-GASZ and FLAG-GASZ proteins when FLAG-P345/401 was co-expressed (Fig. 1F), suggesting that FLAG-P345/401 competitively blocked the self-interaction of full-length GASZ. Taken together, these results support that aa 345 to 401 of GASZ mediates its protein dimerization in the absence of other germ cell-specific proteins.

### GASZ self-interaction is important for its protein stability

**Fig. 2.**
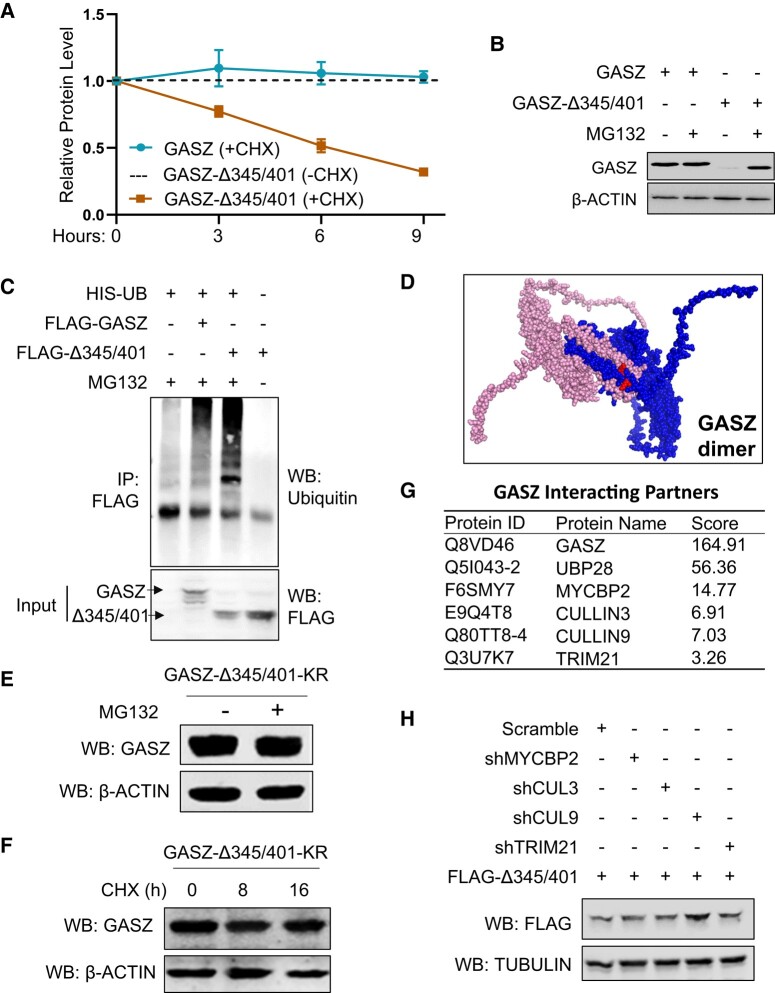
GASZ self-interaction is critical for its protein stability. A) 293T cells were transfected with FLAG-tagged full-length GASZ or GASZ-Δ345/401. CHX has added to the cell culture 36 h post-transfection, and cells were collected at the indicated time periods post CHX treatment. Relative protein levels were determined via grey-scale intensity of protein bands detected by Western Blots with a FLAG antibody from three independent experiments. B) 293T cells were transfected with full-length GASZ or GASZ-Δ345/401. MG132 was added to the cell culture 36 h post-transfection, and cells were then collected 10 h post MG132 treatment for WB. C) 293T cells were cotransfected with HIS-tagged ubiquitin (HIS-UB) and FLAG-tagged full-length GASZ, or FLAG-Δ345/401. Cells were treated with or without MG132 for 10 h and cell lysates were IP using a FLAG antibody for WB with antibodies indicated on the right. D) The protein structure of GASZ dimer (one molecule in pink and the other in blue) was predicted using Alphafold2. The two lysine residues from the blue GASZ protein were labeled with red. E and F) 293T cells were transfected with GASZ-Δ345/401 containing two lysine to arginine mutations. Cells were then treated for 10 h in the absence (−) or presence (+) of MG132, followed by WB (E). Alternatively, cells were treated for indicated hours (h) with CHX before being collected for WB (F). G) List of deubiquitinase and E3-ligases in testicle cells that were co-IP with a GASZ antibody. H) C18-4 cells were cotransfected with FLAG-Δ345/401 and shRNAs against various E3-ligases. Proteins were detected by WB using antibodies indicated on the left. (C, E, F, H) WB, Western Blot.

Compared to the full-length GASZ, we noticed that more plasmid DNA is needed to express an equal level of GASZ-Δ345/401 in transfection assays. This observation suggests that disrupting GASZ self-interaction leads to protein instability. We tested this hypothesis with a cycloheximide (CHX) chasing assay to measure the half-life of GASZ and its interaction deletion mutants. We found that the protein level of full-length GASZ was quite stable, whereas the half-life of GASZ mutant proteins without their self-interaction domain (e.g. GASZ-Δ345/401) was significantly reduced in the presence of CHX, an inhibitor that suppresses new protein synthesis (Figs. 2A and S3A and B) (32).

We further examined whether the GASZ mutant protein was degraded *via* the ubiquitination-proteasome pathway. Using MG132 as a proteasome inhibitor (33), we found significantly increased protein levels of GASZ mutants without their self-interaction domain (e.g. GASZ-Δ345/401), compared to the ones without MG132 treatment (Figs. 2B and S3C). In addition, GASZ-Δ345/401 also showed a higher amount of ubiquitinated proteins than the full-length GASZ (Fig. 2C).

Upon CHX treatment, although deletion of self-interaction domain (e.g. GFP-GASZ-Δ214/401) reduced GASZ protein level, truncated GASZ without the entire bZIP domain (GASZ-Δ252–475) displayed stable protein expression over time (Fig. S3B and C), suggesting that the potential ubiquitination targets are likely the two lysine residues (K420 and K425) from the second α-helix of ZIP domain (Fig. S3D). AlphaFold 2 predicted that these two lysine residues (in red) were embedded within the α-helices of GASZ dimerization interface, which shielded them from direct exposure to ubiquitination for proteasome degradation (Fig. 2D). Indeed, site-directed mutagenesis of these two lysine (K) residues to arginine (R) prevented degradation of GASZ-Δ345401-KR proteins even without MG132 treatment (Figs. 2E and S3E and F). Consistently, we observed that the half-life of these mutant GASZ-Δ345401-KR proteins increased in the presence of CHX (Fig. 2F).

We further identified the potential E3-ligases responsible for GASZ protein degradation in testicular cells using Co-IP coupled with Mass Spectrometry with a GASZ antibody (Fig. 2G). We found deubiquitinase UBP28 and four E3-ligases that were co-IP with GASZ, including MYCBP2, CULLN3 (CUL3), CULLN9 (CUL9), and TRIM21 (Fig. 2G). All of these E3-ligases were highly expressed in both mouse testes and C18-4, an immortalized spermatogonial cell line (Fig. S3G). Among these, only knockdown of CUL9 in C18-4 significantly increased the level of mutant GASZ-Δ345/401 proteins (Figs. 2H and S3H), suggesting that CUL9 is a main player for GASZ protein degradation in the ubiquitination-proteasome pathway.

### GASZ self-interaction is essential for mitochondrial clustering in IMC assembly

**Fig. 3.**
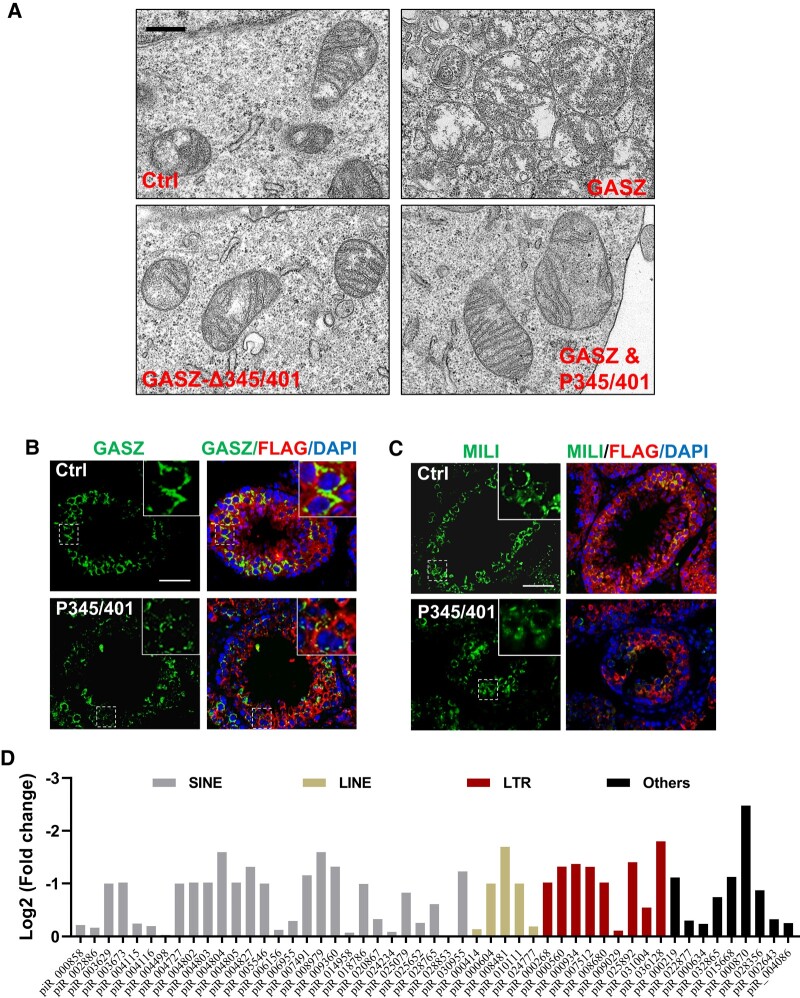
Disrupting GASZ self-interaction dysregulates mitochondrial clustering, IMC formation, and piRNA biogenesis. A) 293T cells were transfected with full-length GASZ, GASZ-Δ345/401, or full-length GASZ together with P345/401. An empty vector (Ctrl) was used as a baseline control. Mitochondria were observed under a transmission electron microscope. Scale bar: 500 nm. B and C) IHF assays were performed on testes collected 8 weeks post viral injection of control FLAG peptide (Ctrl) vs. FLAG-P345/401, using antibodies against FLAG and GASZ (B) or MILI (C), costaining with DAPI. Scale bar: 50 µm. Inserts showed blowup images of representative regions. D) The abundance of piRNAs in spermatogonia upon blocking endogenous GASZ by FLAG-P345/401 was detected using RNA-Seq. Log2 (fold change) was extracted based on averaged read counts of individual piRNAs from FLAG-P345/401 vs. FLAG control group.

To understand whether GASZ self-interaction contributes to mitochondrial clustering and IMC assembling, we enforced the expression of full-length GASZ, GASZ-Δ345/401, or the peptide aa 345–401 (P345/401) with full-length GASZ in 293 T cells that contain no other germ cell-specific proteins. We then examined mitochondrial aggregation under each circumstance by TEM. Compared to the empty vector control, we found that full-length GASZ promoted mitochondrial clustering (Figs. 3A and S4A), consistent with our previous findings (23). The mitochondrial clustering was however diminished when GASZ interaction domain was deleted, or in cells co-expressing full-length GASZ and interaction-blocking peptide, P345/401 (Figs. 3A and S4A). Thus, these data support that GASZ relies on its self-interaction to cluster mitochondria.

Because the peptide that was produced from aa 345–401 of GASZ could efficiently block the self-interaction of wildtype GASZ proteins, we next used it to examine whether the disrupting GASZ self-interaction had any functional consequences in IMC assembly of germ cells. We injected lentivirus expressing FLAG-P345/401 via efferent ducts into one testis of adult mice to block the self-interaction of endogenous GASZ proteins. In the contralateral testis from the same mouse, we introduced virus solely encoding a FLAG peptide as a control. After 8 weeks postinjection, we observed that mitochondrial staining by TOMM20 displayed smaller and more diffused patches in germ cells (Fig. S4B), compared to control testes. We also examined the IMC integrity by immunostaining with antibodies against GASZ or MILI (also called PIWIL2/piwi-like RNA-mediated gene silencing 2), two IMC proteins. Indeed, we observed that the staining patterns of these proteins were reduced into much smaller patches in the majority of FLAG-P345/401+ germ cells than in controls (Fig. 3B and C), suggesting that disrupting GASZ self-interaction reduces mitochondrial clustering and in turn destabilizes existing IMC assembly in germ cells.

Because IMC provides a subcellular compartment for piRNA biogenesis, we assessed the piRNA levels upon blocking endogenous GASZ self-interaction. Primarily cultured neonatal spermatogonia were introduced with virus expressing a control FLAG peptide or FLAG-P345/401. The total RNAs were then collected for small noncoding RNA-seq. We found that the total abundance of mature piRNAs between 26 and 32 nt was detectably decreased upon enforced expression of FLAG-P345/401 (Fig. S5A and Table S1), suggesting that GASZ self-interaction is crucial for its known function in piRNA biogenesis. Retrotransposon sequences often serve as sources to produce pre-pachytene piRNAs. We thus analyzed the abundance of these retrotransposon-derived piRNAs from our RNA-seq data. Indeed, many piRNAs from transposable elements including SINEs (short interspersed nuclear elements), LINEs (long interspersed repetitive DNAs), and LTR (long terminal repeats) were downregulated in FLAG-P345/401 group when GASZ protein interaction was blocked (Fig. 3D). Independent experiments with real-time RT-PCR further confirmed these results. We found that in the spermatogonia with enforced expression of full-length GASZ, the piRNA levels were increased, while FLAG-P345/401-expressing spermatogonia displayed a significantly lower level of piRNAs (Fig. S5B). Taken together, our data suggest that GASZ self-interaction is essential to maintain its protein stability in germ cells, which in turn stabilizes IMC structure for piRNA biogenesis.

### Disrupting GASZ self-interaction impairs spermatogenesis

**Fig. 4.**
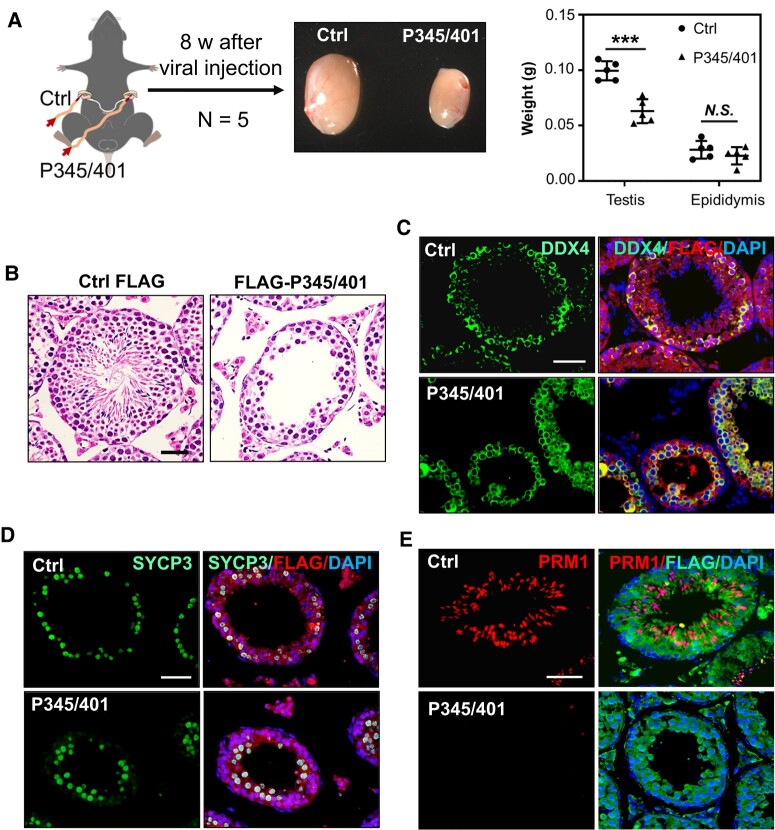
Disrupting GASZ self-interaction impairs spermatogenesis. A) A virus expressing FLAG-P345/401 (P345/401) was injected into one testis of adult mice via efferent ducts, while virus expressing a control FLAG peptide (Ctrl) was into the contralateral testis of the same mouse. Representative images of testes 8 weeks (w) post viral injection were shown. The weights of testis and epididymis from five mice were presented as mean ± SEM on the right. ***P < 0.001. N.S., no significance. B) Histology of testes with virus expressing a control FLAG peptide vs. FLAG-P345/401 8 weeks postviral injection. C and E) IHF assays were performed on testes collected 8 weeks post viral injection of FLAG peptide vs. FLAG-P345/401 using antibodies against FLAG and DDX4 (C), SYCP3 (D), or PRM1 (E), counterstained with DAPI. B–E) Scale bars: 50 µm.

*Gasz* knockout or MLS mutant during the embryonic stage leads to early meiotic arrest in spermatogenesis. However, the role of GASZ or its self-interaction in maintaining germ cell development during adulthood remains unexplored. Upon injecting FLAG-P345/401 into seminiferous tubules to disrupt endogenous GASZ self-interaction, we observed that the FLAG-P345/401-treated testes were smaller than the control testes (Fig. 4A), indicating reduced germ cell development. Histological analyses further revealed that while the spermatogonia and spermatocytes remained largely intact in the testis with the blocking peptide injection, they contained significantly fewer elongated spermatids (Fig. 4B). Elongated spermatids were readily detectable in more than 95% of seminiferous tubules in control testis. In comparison, about 75% of seminiferous tubules in FLAG-P345/401 treated mice contained no spermatids. We confirmed these results by immunohistofluorescence (IHF) analyses using antibodies against DDX4 that is expressed in germ cells from spermatogonia to spermatids (Figs. 4C and S5C), SYCP3 (spermatocytes, Fig. 4D), and PRM1 (haploid spermatids, Figs. 4E and S5D). SYCP3+ spermatocytes were largely unaffected, but fewer PRM1+ spermatids were detected in the seminiferous tubules with FLAG-P345/401 injection (Figs. 4D and E and S5C and D). These data strongly support a critical requirement of GASZ self-interaction for proper spermatid formation in adulthood.

### Impaired spermatid formation due to blocked GASZ self-interaction can be recovered

**Fig. 5.**
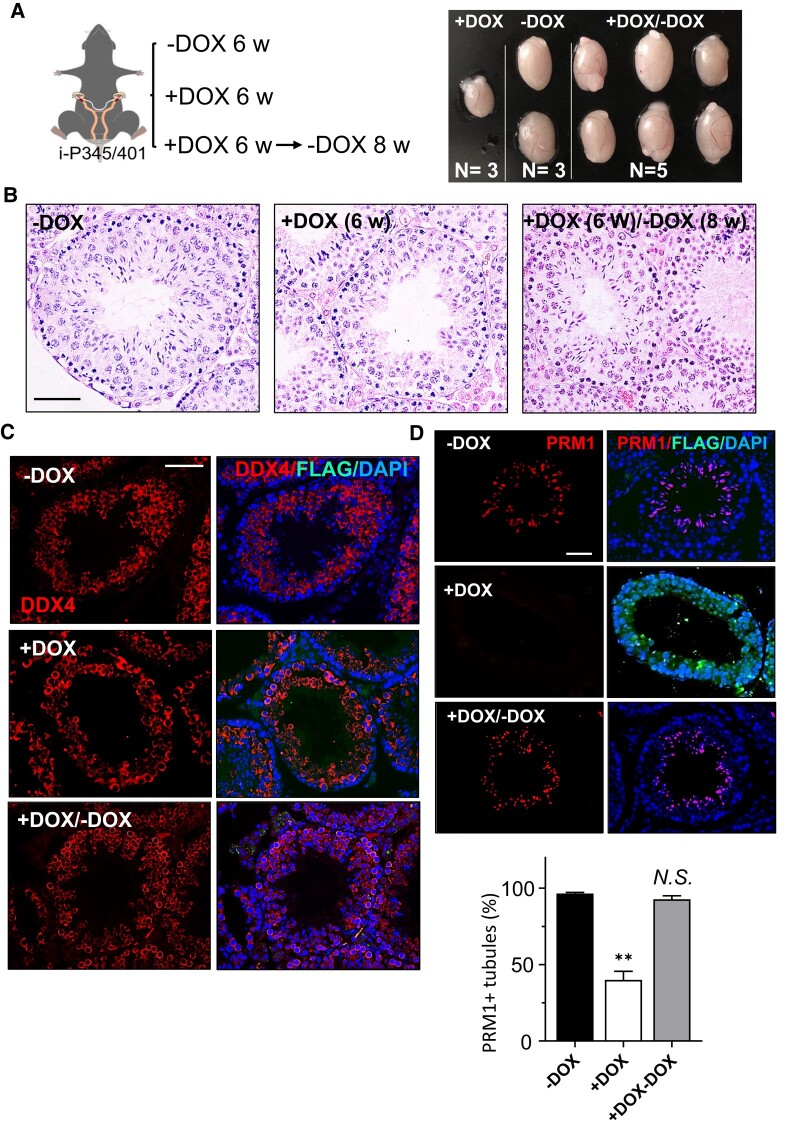
Disrupting GASZ self-interaction reversibly blocks spermatid formation in adult mice. A) A virus expressing a DOX-inducible FLAG-P345/401 was injected into testes via efferent ducts, and mice were fed with normal water (−) or with DOX-containing water (+) for indicated weeks. Histology (B) and IHF were performed using antibodies against FLAG and DDX4 (C) or PRM1 (D), counterstained with DAPI. D) Percentages of seminiferous tubules containing PRM1 + spermatids were calculated based on ∼100 randomly selected seminiferous tubule sections per mouse. Data were represented as mean ± SEM from three mice per group. **P < 0.01. *N.S.,* no significance. B–D) Scale bar: 50 µm.

Because FLAG-P345/401 mainly blocked haploid spermatid formation in adult mice, we next examined whether impaired germ cell development due to disrupted GASZ self-interaction was reversible. We injected the testes of adult mice with virus containing a doxycycline (DOX)-inducible FLAG-P345/401. Mice were then fed with DOX-containing water to induce the expression of this blocking peptide for 6 weeks, using mice on normal water as controls. Similar to our observation when injecting the constitutively expressed FLAG-P345/401, we found smaller testes in DOX-treated mice, indicating disrupted spermatogenesis. We then switched DOX to normal water (Fig. 5A, left panel). Strikingly, 8 weeks after DOX withdrawal, we found that the testes returned to normal size (Fig. 5A, right panel and Fig. S5E), suggesting that blocked spermatogenesis is reversible. We further confirmed these results using both histology (Fig. 5B) and IHF (Fig. 5C and D). We found DDX4+ spermatogonia and spermatocytes remained comparable across three groups (Fig. 5B and C). There were few spermatids in DOX-treated testes, but PRM1+ haploid cells were readily recovered eight weeks after DOX withdrawal (Fig. 5D). In summary, our data suggest that the disruption of GASZ self-interaction reversibly blocked spermatogenesis in adult mice.

## Discussion

It is generally believed that multiple proteins form a stable complex to cluster mitochondria into IMC, creating a unique infrastructure to support coordinated piRNA biogenesis and other biological functions in male germ cells (16–24). However, most key IMC players including MILI, MIWI (also called PIWIL1/piwi-like RNA-mediated gene silencing 1), and TDRD1, do not contain MLS (4, 19, 34), and thus rely on other mitochondrial proteins to recruit them to IMC. Although TDRKH is a germ cell-specific mitochondrial protein at IMC, its deletion during the embryonic stage does not affect IMC integrity (15, 35). It remains unclear how IMC forms in embryonic prospermatogonia and postnatal spermatogonia to support spermatogenesis. GASZ is highly expressed at embryonic day 13.5 (29), and is the only known germ cell-specific mitochondrial protein that is expressed when IMC starts to appear and affects IMC integrity when deleted from embryonic germ cells (23, 27). In this study, we found that GASZ formed dimers and were able to cluster mitochondria in somatic cells when no other germ cell-specific proteins were present. When the self-interaction of GASZ proteins was disrupted in adult germ cells, IMC was destabilized. These data strongly suggest that GASZ self-interaction initiates and maintains mitochondrial clustering for IMC assembling. However, in primarily cultured neonatal spermatogonia, piRNA biogenesis was only moderately reduced upon disrupted GASZ self-interaction. It is thus possible that GASZ self-interaction has additional regulatory effects beyond piRNA production. We previously demonstrated that GASZ also interacted with the mitochondrial proteins, MFN1 and MFN2, to promote mitochondrial fusion and metabolism (23). We found in this study that the pro-fusion function of GASZ might also depend on its protein self-interaction (Fig S4C). Interestingly, other players in piRNA biogenesis and male germ cell development, such as MitoPLD, were also reported in regulating mitochondrial fusion (25, 26). The functional relationship between IMC and mitochondrial activities as well as its impact on male germ cell development remains unclear and warrants further investigation.

IMC is a subcellular compartment in germ cells with a stable presence from embryonic prospermatogonia to postnatal spermatocytes (5, 6). IMC protein stability and interactions are crucial for maintaining such a stable structure without any limiting membrane. We revealed that GASZ self-interaction was critical for maintaining its protein stability. AlphaFold 2 analyses predicted that two α-helices of the basic leucine zipper formed a fork-like structure to lock GASZ into dimers (Figs. 2D and S2B). As a result, the lysine residues of the second α-helix are hidden inside the GASZ protein dimers and protected from proteosome-mediated degradation. Theoretically, the best model to determine the physiological role of GASZ self-interaction during germ cell development is to use a genetically modified mouse model, in which the wildtype GASZ is replaced by a GASZ mutant with its self-interaction domain deleted. In reality, GASZ proteins without their self-interaction domain are extremely unstable due to both abolished self-interaction and altered protein structure. This makes it experimentally impossible to distinguish the functional impacts of interaction mutants from those of *Gasz* knockout. Therefore, instead of generating a GASZ mutant without its self-interaction motif, we utilized the lentiviral injection approach with a peptide that competitively blocked GASZ self-interaction. In this case, although endogenous GASZ protein level was moderately decreased, a large fraction of GASZ proteins remained intact. Thus, the phenotype we have observed in these mice is not entirely the same as that in *Gasz* knockout, where *Gasz* is completely removed in gonocytes without IMC formation since the embryonic stage. Nevertheless, the existing IMC was diminished with subsequent impaired spermatid formation upon disrupted GASZ self-interaction, thereby supporting the critical role of GASZ self-interaction in stabilizing IMC structure for proper germ cell development in adulthood.

Unintended pregnancies make up about 30% of total pregnancies in the U.S. each year. While there are multiple reversible contraceptive options for women, condoms and physical withdrawal are the only options for men, however, with high failure rates of 13 and 20%, respectively (36–38). Thus, there is an urgent need to develop safer and more effective male contraceptives. In contrast to hormonal male contraception in clinical trials, which stops sperm production by inhibiting the hypothalamic—pituitary axis, nonhormonal contraceptive usually targets proteins that specifically regulate germ cell development and functions, thereby avoiding potential side effects associated with whole-body hormone treatment (36–38). This study identified GASZ self-interaction as a new target for developing nonhormonal reversible male contraceptives. Notably, GASZ proteins share high sequence similarity across mammalian species, with ∼91% identical residues from aa 345 to 401 (the critical region for GASZ self-interaction) between humans and mice (Fig. S2B and C). We previously demonstrated that GASZ was expressed only in testes and had a functional conservation in promoting germ cell development in both humans and mice (29). When GASZ self-interaction was abolished in adult male germ cells, spermatogonia, and spermatocytes were largely unaffected, but spermatid formation was blocked, and few spermatids were detected in the seminiferous tubules. In addition, this blockage of germ cell development is reversible. Spermatogenesis could be recovered after GASZ self-interaction was resumed, providing a proof-of-concept for targeting IMC protein interactions in developing new male contraceptives. Taken together, this study reveals a critical mechanism by which the piRNA processing machinery is correctly assembled to support proper germ cell development, critically informing mechanistic causes of male infertility and an alternative strategy for developing reversible nonhormonal male contraceptives.

### Experimental materials and methods

#### Animal experiments

All animal experimental procedures were approved by the Institutional Animal Care and Use Committee at Michigan State University and conducted in compliance with the regulatory guidelines. Lentiviral injection *via* the efferent duct was performed as described previously (39). Briefly, ∼5–10 µL of lentivirus with trypan blue were injected into the seminiferous tubules of the testes from ∼5-week-old C57BL/6 mice (the Jackson Lab). Testes were analyzed at 6–14 weeks post injection. For DOX-inducible expression in mouse testes, water was changed every week with 400 µg/mL of freshly made DOX.

#### Histology study and IHF assays

For histology analyses, testes were fixed in Bouin's solution (Sigma-Aldrich, HT10132), followed by dehydration, embedded in paraffin, and processed into 4 µm sections for hematoxylin (Sigma-Aldrich, MHS16) and eosin (Sigma-Aldrich, HT110216) staining. Images were obtained with a Leica microscope (Leica, DM4000B). IHF was performed as previously described (23). Briefly, testes were fixed with 4% paraformaldehyde in PBS (Phosphate Buffered Saline) at 4°C overnight and were then embedded in paraffin. The 4 µm-thick testicular sections were incubated with primary antibodies followed by washing three times with PBS containing 0.2% Tween-20. Nuclei were stained with DAPI (4′,6-diamidino-2-phenylindole) after blotting with fluorochrome-conjugated 2nd antibodies. Images were obtained with a Leica microscope (Leica, DM4000B) or a Leica confocal microscope (Leica TCS/SP5). Primary antibodies used in this study: FLAG (Sigma-Aldrich, F7425; Sigma-Aldrich, F1804), DDX4 (Abcam, ab196708/ab13840), SYCP3 (Abcam, ab15093/ab97672), PRM1 (Briar Patch Biosciences, MAb-Hup1N-150), MILI (Cell Signaling Technology, 5940S), and TOMM20 (Abcam, ab186734). GASZ antibody was generated from rabbits using GST (Glutathione-S-Transferase)-tagged full-length GASZ as an immunogen, and its specificity was confirmed in testes from wildtype and GASZ-ΔMLS mutant mice. Fluorochrome-conjugated second antibodies (Alexa Fluor 488 AffiniPure Goat Anti-Mouse IgG, Cat#115-545-003; Alexa Fluor 594 AffiniPure Goat Anti-Rabbit IgG, Cat#111-585-003) were from Jackson ImmunoResearch Laboratories.

#### Plasmid construction for gene overexpression and knockdown

*Gasz* cDNA was obtained and described in our previous study (23). The expression vector, pET-6HIS-MBP, was a kind gift from Dr. Jian Hu at Michigan State University. The truncated mutants or blocking peptide of the *Gasz* were subcloned into pEGFP-c1, pll3.7 fused with FLAG tag, or pcDNA3.1 in frame with the N- or C-terminal half of YFP. The K to R mutations in GASZ protein were generated by PCR-based mutagenesis. For gene knockdown experiments, the sense oligos were designed as 5′T-(GN18)-(TTCAAGAGA)-(18NC)-TTTTTTC, with the complimentary antisense oligos plus additional nucleotides at the 5′ ends to generate *XhoI* overhang. Annealed oligos were cloned into pll3.7-U6 between *HpaI* and *XhoI*. All plasmids were sequenced to confirm the correct inserts and mutations. Primers for cloning, mutagenesis, and gene knockdown are listed in Table S2.

#### Cell culture, transfection, and viral infection

HeLa, 293T, and C18-4, cells were cultured in DMEM (Dulbecco's Modified Eagle Medium) (Gibco) with 10% fetal bovine serum (Gibco) and transfected with plasmids using polyethylenimine (Sigma) for ectopic gene expression. Mouse spermatogonia were cultured according to published protocols (40, 41). For viral infection of spermatogonia, lentivirus was packaged in 293 T cells and concentrated by Amicon Ultra-50 Centrifugal Filter Unit (Sigma, UFC9100) into 6 × 10^7^/mL. Cell suspension was mixed with concentrated virus with MOI (multiplicity of infection) at 2 and 5 µg/mL polybrene. Cell medium was changed at 20 h postinfection.

#### Transmission electron microscopy

TEM was performed according to published protocols (23). Briefly, 293 T cells were washed in PBS and fixed in 2.5% glutaraldehyde. Cells were transferred into 1.5 mL centrifuge tubes, and cell pellets were embedded into 2% low-melting agarose gel for subsequent osmification, dehydration, and embedding into the resin. Embedded samples were cut into 70 nm sections by Ultra microtome (Leica UC7) for further staining with uranyl acetate and lead citrate. Images were obtained using a Hitachi HT7800 electron microscope.

#### Immunofluorescence assays

Cells were seeded on cover slides and fixed in 4% paraformaldehyde. Immunofluorescence (IF) was performed using a GFP antibody (Abcam, ab183734) or antibodies described in IHF. The fluorochrome-conjugated second antibodies (Jackson ImmunoResearch Inc.) were used with 1:200 dilution. Images were acquired using a Leica confocal microscope (Leica TCS/SP5).

#### Protein purification and native PAGE

BL21 bacteria (New England Biolab, C2527) were transformed with HIS-MBP-GASZ-ΔMLS expressing plasmid. Protein expression was induced with 0.2 mM isopropyl-β-D-thiogalactopyranoside/IPTG (Thermofisher, 15529019) when OD600 of culture reached 0.4. Induction continued at room temperature for 20 h before harvesting. Proteins were purified using HisPur Ni-NTA Resin (ThermoFisher Scientific, 88221) in 50 mM Tris (pH = 7.6), 300 mM NaCl, and 0.1% DDM (n-Dodecyl-B-D-Maltoside). After removing imidazole using an Amicon centrifugal filter (Sigma), proteins were loaded onto a Superdex Increase 200 column (GE Healthcare) that had been equilibrated with 50 mM Tris (pH 7.6), 100 mM NaCl, and 0.01% DDM. For native PAGE, proteins were prepared in a nonreducing and nondenaturing sample buffer (50 mM Tris, pH = 7.6, 100 mM NaCl, and 0.01% DDM) and mixed with nondenaturing loading buffer (62.5 mM Tris–HCl, pH = 6.8, 25% glycerol, 1% bromophenol blue) for electrophoresis. The gel was stained with Coomassie Brilliant Blue R-250 Staining Solution (Biorad, 1610436) before blotting with a GASZ antibody. Images were acquired using Li-COR Odyssey system (LI-COR Biosciences).

#### Western blot, co-IP, and mass spectrometry

Western blots were performed according to standard protocols. Primary antibodies were used in this study: Ubiquitin (Abcam, ab134953), HIS (ThermoFisher Scientific, MA1-135); β-actin (Santa Cruz Biotechnology, sc-47778), β-tubulin (Santa Cruz Biotechnology, sc-5274). Other antibodies were described above in the IHF/IF sections. The fluorochrome-conjugated 2nd antibodies were used at 1:10,000 dilution (LI-COR Biosciences, P/N: 926-49020; LI-COR Biosciences P/N: 926-68070). The fluorescent signals against targeted proteins were detected using the Li-COR Odyssey system. For Co-IP, immunoprecipitation was performed as described previously (23). Briefly, cells were harvested in lysis buffer (50 mM Tris, pH7.6, 300 mM NaCl, and 1% Triton X-100 with protease inhibitor cocktail) and sonicated at 4 °C. Cleared supernatant was incubated with FLAG affinity beads (Sigma, A2220), GFP magnetic beads (Bio-linkedin, L1016), or HisPur Ni-NTA Resin (ThermoFisher Scientific, 88221). The IP proteins were detected using Western blot (WB). For IP–mass spectrometry (MS), testes were homogenized in lysis buffer for sonication, and the cleared supernatant was incubated with a GASZ antibody, followed by a pull-down with protein A/G-agarose beads (Santa Cruz Biotech, sc-2003). IP proteins were washed by lysis buffer and washing buffer (50 mM Tris, pH7.6, 100 mM NaCl) before detection with LC (Liquid chromatography)/MS using the Q Exactive Orbitrap Mass Spectrometers (ThermoFisher Scientific).

#### Small noncoding RNA-sequencing and bioinformatic analyses

Biological triplicates of spermatogonia expressing either the control FLAG or FLAG-P345/401 peptides were collected for constructing small RNA libraries with GenSeq Small RNA Library Prep Kit according to the manufacturer's instruction (GenSeq, Inc.). Briefly, 3′ and 5′ adaptors were successively ligated to RNAs, which were reversely transcribed into cDNA, followed by PCR amplification. The barcoded cDNA libraries were size-selected for piRNA fractions (20–40 nt) before sequencing with a NovaSeq platform (Illumina). The adaptor sequences were trimmed from raw data using a cutadapt software, and the trimmed sequences (≥15 nt) were aligned to known piRNAs from piRNABank using the Novoalign software (v3.02.12) allowing for one mismatch or less. The read counts per million aligned piRNAs were normalized based on total miRNA counts. The piRNA targets were predicted using the miranda software (v3.3a).

#### RT-PCR, PCR, and real-time PCR

Total RNAs were extracted with TRIzol (ThermoFisher) and reverse-transcribed using a PrimeScrip RT reagent Kit (TAKARA, RR037A) according to manufacturers' instructions. PCR and real-time PCR were performed as described previously (42). Primers used in this study are listed in Table S2.

#### AlphaFold 2 prediction of protein structure and protein interactions

The full-length protein sequence of mouse GASZ (NP_076218.3) was downloaded from NCBI (National Center for Biotechnology Information). Structures of GASZ and GASZ dimers were predicated using the AlphaFold 2 at Google Colab following the published protocol (31). Models were generated with the following parameters: rank number = 5, multiple sequence alignment mode = MMseqs2, pair_mode = Unpaired + paired, and number_recycles = 3. The top five models were ranked based on predicted local distance difference test scores per-residue. These models were analyzed by PyMOL software for visualization of the interacting interface. All top five models showed a similar structure of GASZ protein and interaction interface of aa 345–401 in GASZ dimer.

#### Statistical analysis

Data were presented as mean ± SEM. All experiments were performed independently three times or more unless otherwise stated. Statistical comparisons of between-group means were performed using unpaired Student's t test and the Prism Graphic software.

## Supplementary Material

Supplementary material is available at *PNAS Nexus* online.

## Supplementary Material

pgad480_Supplementary_Data

## Data Availability

Unique experimental materials will be provided upon request. Raw small RNA-seq data were uploaded to SRA (Sequence Read Archive) repository and accessible to public: https://dataview.ncbi.nlm.nih.gov/object/PRJNA1031556?reviewer=fp2ptl2u678niuldr3heana01m.
