# Supplementary material for: GASZ self-interaction clusters mitochondria into the intermitochondrial cement for proper germ cell development
Source: PNAS Nexus. 2024 Jan 2;3(1):pgad480. doi: 10.1093/pnasnexus/pgad480 (PMC10781510; doi:10.1093/pnasnexus/pgad480)
Supplement: pgad480_Supplementary_Data [file pgad480_supplementary_data.zip › PNASNEXUS-PNASNEXUS-2023-00879RR-s01.pdf]

Supplemental Information

Supplemental Experimental Materials and Methods

Supplemental Figure Legends

Fig. S1. GASZ proteins self-interact to aggregate mitochondria. (**A-B**) Size-exclusion chromatograph analyses and purification of GASZ proteins (black curves) using protein standards with known molecular weight (green curves). Protein standard 1: Thyroglobulin (670 KD); 2: Globulin (158 KD); 3: BSA (66 KD); 4: Ovalbumin (44 KD); 5: Myoglobin (17 KD); and 6, Vitamin B12 (1 KD). (**A**) GDN-solubilized HIS-MBP-GASZ-ΔMLS. (**B**) DDM-solubilized GASZ-ΔANK. The theoretical molecular weight of GASZ-ΔANK is ~28 KD. DDM is known to form micelles at ~42 KD. The DDM-solubilized GASZ-ΔANK monomer is ~70 KD. The major peak of DDM-solubilized GASZ-ΔANK was close to 158 KD, suggesting that dimers were formed. (**C**) FLAG-tagged GASZ, GASZ-ΔMLS, or GASZ-Δ345/401 proteins were expressed in HeLa cells. Protein subcellular localization was visualized by IF using antibodies against TOMM20 (red) and FLAG (green), counterstained with a nucleus staining dye DAPI. Representative cell images were shown. Scale bar: 25 μm.

Fig. S2. The aa residues 351 to 401 of GASZ are responsible for its protein self-interaction. (**A**) Bimolecular fluorescence complementation assays were performed on HeLa cells that were transfected with transgenes indicated at the top of each image. Green fluorescence was visualized 48 hours post transfection. Scale bar: 100 μm. (**B**) GASZ protein dimerization predicted by AlphaFold 2 in google Colab, visualized by PyMOL, with one GASZ molecule in blue, another in grey. Interacting amino acid residues were colored red. (**C**) GASZ protein is highly conserved across mammalian species, and 91% of aa 345-401 are identical between human and mouse.

Fig. S3. GASZ self-interaction is critical for its protein stability. (**A-B**) 293T cells were transfected with FLAG-tagged full-length GASZ or various GASZ deletion mutants. CHX was

added to the cell culture 36 hours post transfection, and cells were collected at indicated hour (h) during CHX treatment for Western Blots using FLAG and β-ACTIN antibodies. (**C**) 293T cells were transfected with full-length GASZ or various GASZ deletion mutants. MG132 was added to cell culture 36 hours post transfection, and cells with or without MG132 treatment were collected 10 hours post treatment for Western Blot analyses. Antibodies used in Western Blot were indicated on the right. (**D**) Protein sequences of GASZ, with ANK in purple, SAM in blue, and bZIP in red. The two lysine (K) residues in 2nd helix of bZIP domain are labeled as underlined bold green fonts. (**E**) 293T cells were co-expressed with HIS-ubiquitin and full-length GASZ, or GASZ with lysine to arginine mutations, and/or P345/401. Ubiquitinated GASZ proteins were collected *via* Ni-NTA resin purification, followed by Western Blot using a GASZ antibody. (**F**) 293T cells were transfected with full-length GASZ, or mutant GASZ with lysine to arginine mutations, and/or FLAG-tagged peptide containing aa 345-401 of GASZ, followed by MG132 treatment (+). Protein levels were detected by Western Blot analyses with antibodies indicated on the left. (**G**) RT-PCR analyses of E3-ligases on testes from day 14 mice and C18-4 spermatogonial cell line. (**H**) C18-4 cells were con-transfected with FLAG-GASZ-Δ345/401 and shRNAs against various E3-ligases. Gene knockdown was confirmed by real-time RT-PCR analyses on target E3-ligase. (**G-H**) Data were represented as mean ± SEM from three biological replicates. *: p<0.05; **: p<0.01; ***: p<0.001. (**B, C, E, F**) WB: Western Blot.

Fig. S4. Disrupting GASZ self-interaction reduces mitochondrial aggregation and destabilizes IMC. (**A**) 293T cells were transfected with full-length GASZ, GASZ-Δ345/401, or full-length GASZ together with P345/401. Empty vector was used as a control. Mitochondria were observed using TEM. Red circled regions show the clustered mitochondria. (**B**) Virus expressing FLAG-P345/401 was injected into one testis *via* efferent ducts, with control FLAG peptide expressing virus into the contralateral testis of the same mouse. Testes were collected 8 weeks post viral injection, and IHF were performed with antibodies against FLAG and TOMM20,

counterstained with DAPI. Inserts showed blow-up images of representative regions. Scale bar: 50 μm. (**C**) Full-length GASZ or GASZ-Δ345/401 (Δ345/401) were transfected into HeLa cells, and mitochondria were stained with TOMM20. Representative cell images were shown on the right. Cell types were classified into fragmented (with smaller mitochondria), tubular (with elongated mitochondria), and intermediate (with mixed types of mitochondria), based on the dominant type of mitochondria in the cell. The percentages of cell types were calculated from 100 randomly selected cells per group and presented as mean ± SEM from three biological replicates. *: $p<0.05$; **: $p<0.01$; *N.S.:* no significance.

Fig. S5. Disrupting GASZ self-interaction dysregulates piRNA biogenesis and spermatid formation. (**A**) Spermatogonia were infected with lentivirus expressing either control FLAG or FLAG-P345/401 peptides, and their piRNA expression profiles were analyzed using small RNA-seq. Graph shows the total normalized tag counts of piRNAs per million aligned RNAs (TPM) grouped by their lengths (nt). N = 3. (**B**) Spermatogonia were infected with lentivirus expressing full-length GASZ, control FLAG peptide, or FLAG-P345/401. Real-time RT-PCR analyses were performed to analyze the relative expression levels of either piRNA. Data were presented as mean ± SEM from 3 independent experiments. *: $p<0.05$; **: $p<0.01$; ***: $p<0.001$. *N.S.:* no significance. (**C-D**) Virus expressing FLAG-P345/401 (P345/401) was injected into one testis of adult mice *via* efferent ducts, while virus expressing a control FLAG peptide (Ctrl) into the contralateral testis of the same mouse. IHF assays were performed on testes collected 8 weeks post viral injection of FLAG peptide *vs* FLAG-P345/401 using antibodies against DDX4 (**C**) or PRM1 (**D**), counterstained with DAPI. The percentages (%) of signal positive seminiferous tubules from 4 testicle sections of 2 mice were presented as mean ± SEM. *: $p<0.05$; *N.S.:* no significance. (**E**) Virus expressing a DOX-inducible FLAG-P345/401 was injected into testes *via* efferent ducts, and mice were fed with normal water (-) or with DOX containing water (+) for indicated weeks (Fig.

5). The weights of testis from 3 to 5 mice were presented as mean ± SEM. ***: $p<0.05$. *N.S.:* no significance.

Supplemental Table S1 The list of piRNA detected by small RNA-seq on spermatogonia infected with virus expressing a control FLAG or FLAG-P345/401.

Supplemental Table S2 The list of primers used in this study.

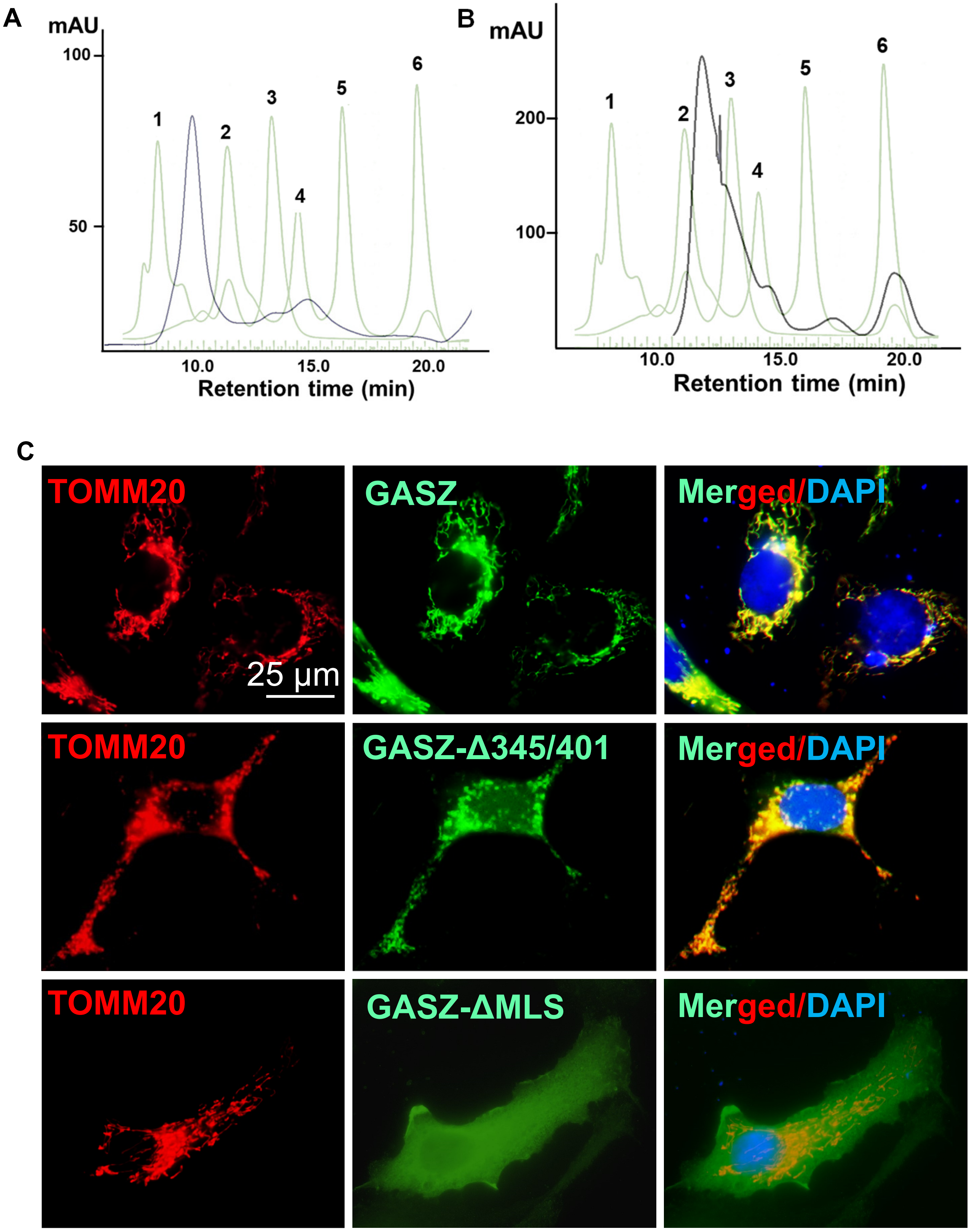

Miao et al., Supplemental Fig. S1

A

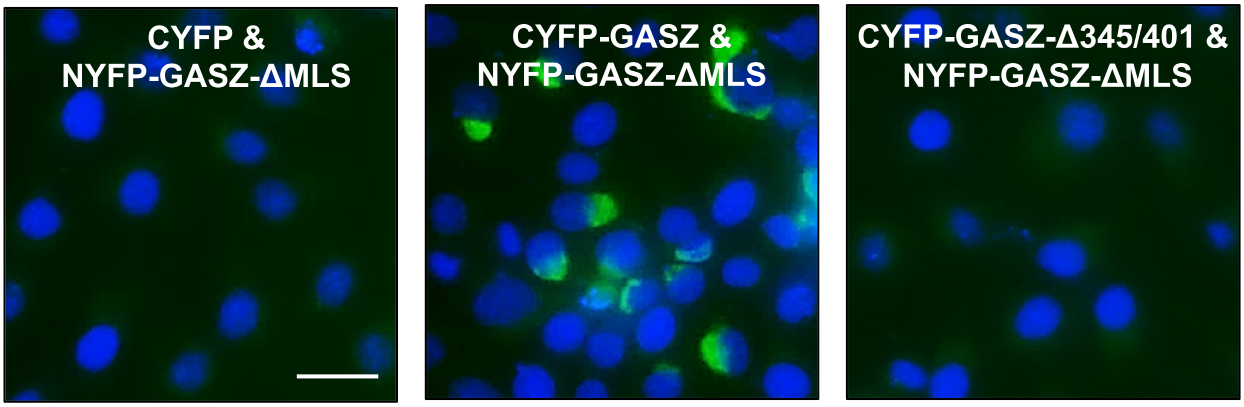

B

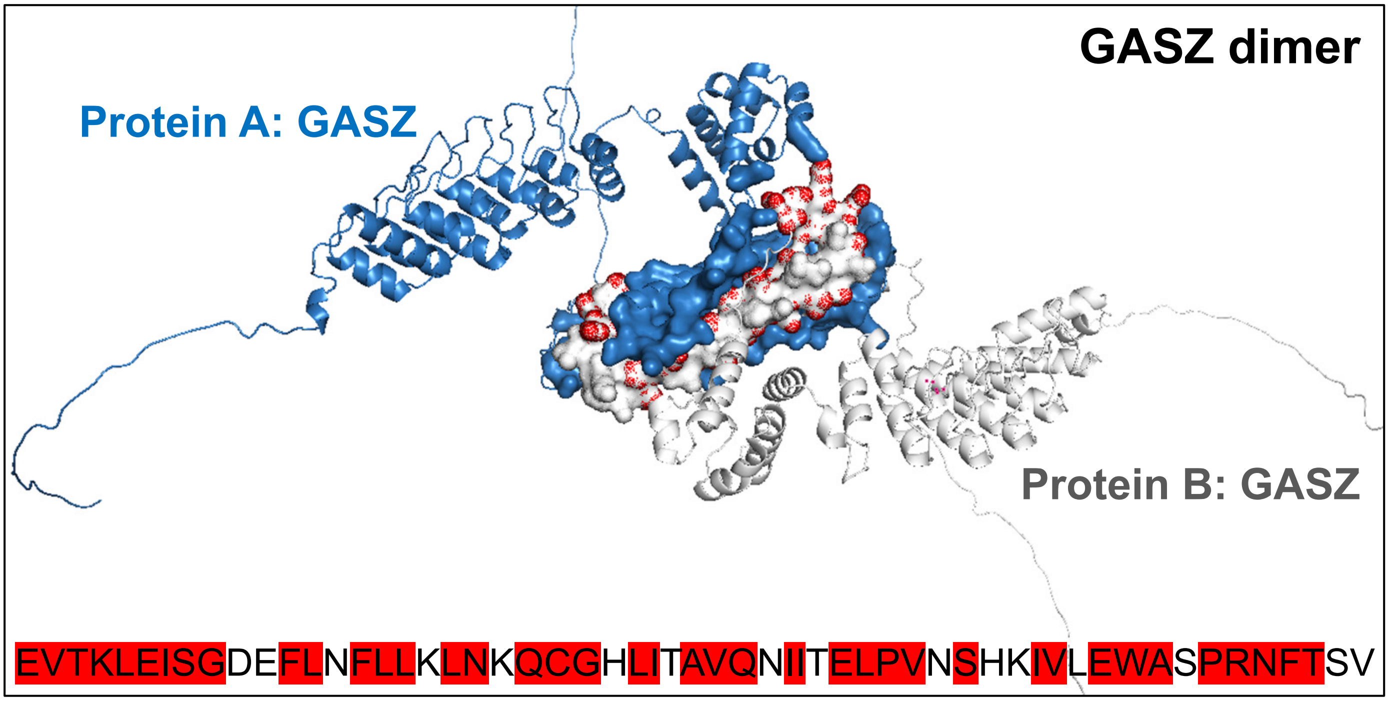

C

Mouse	EVTKLEISGDEFLNFLLKLNKQCGHLITAVQNIITELPVNSHKIVLEWASPRNFTSV
Human	EETKLEISGDEFLNFLLKLNKQCGHLITAVQNVITELPVNSQKITLEWASPQNFTSV
Cattle	EVAKLEISGDEFLNFLLKLNKQCGHLITAVQNIITELPVNSHKIVLEWASPRNFTSV
Pig	EVAKLEISGDEFLNFLLKLNKQCGHLITAVQNIITELPVNSHKIVLEWASPRNFTSV

Miao et al., Supplemental Fig. S2

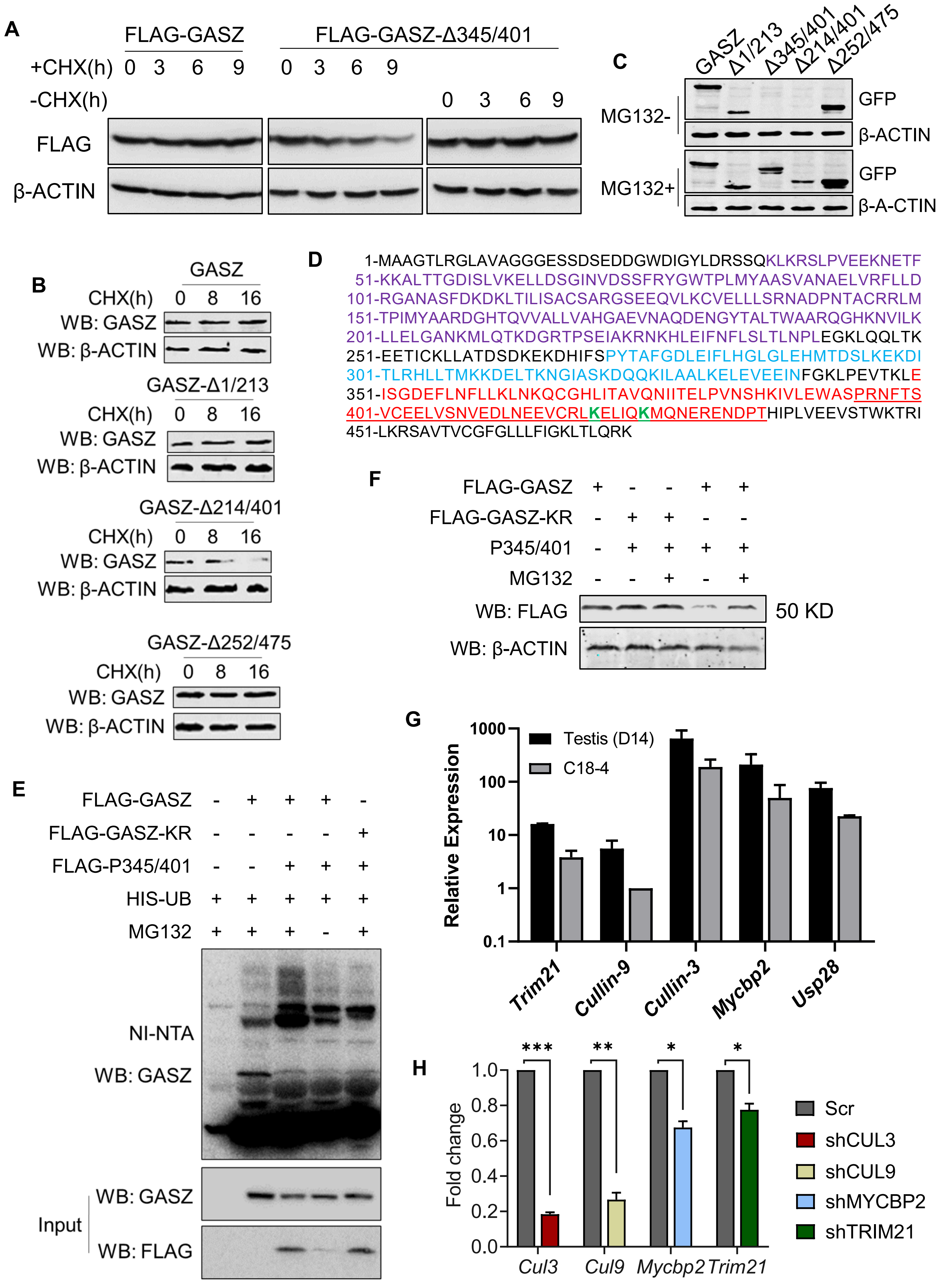

Miao et al., Supplemental Fig. S3

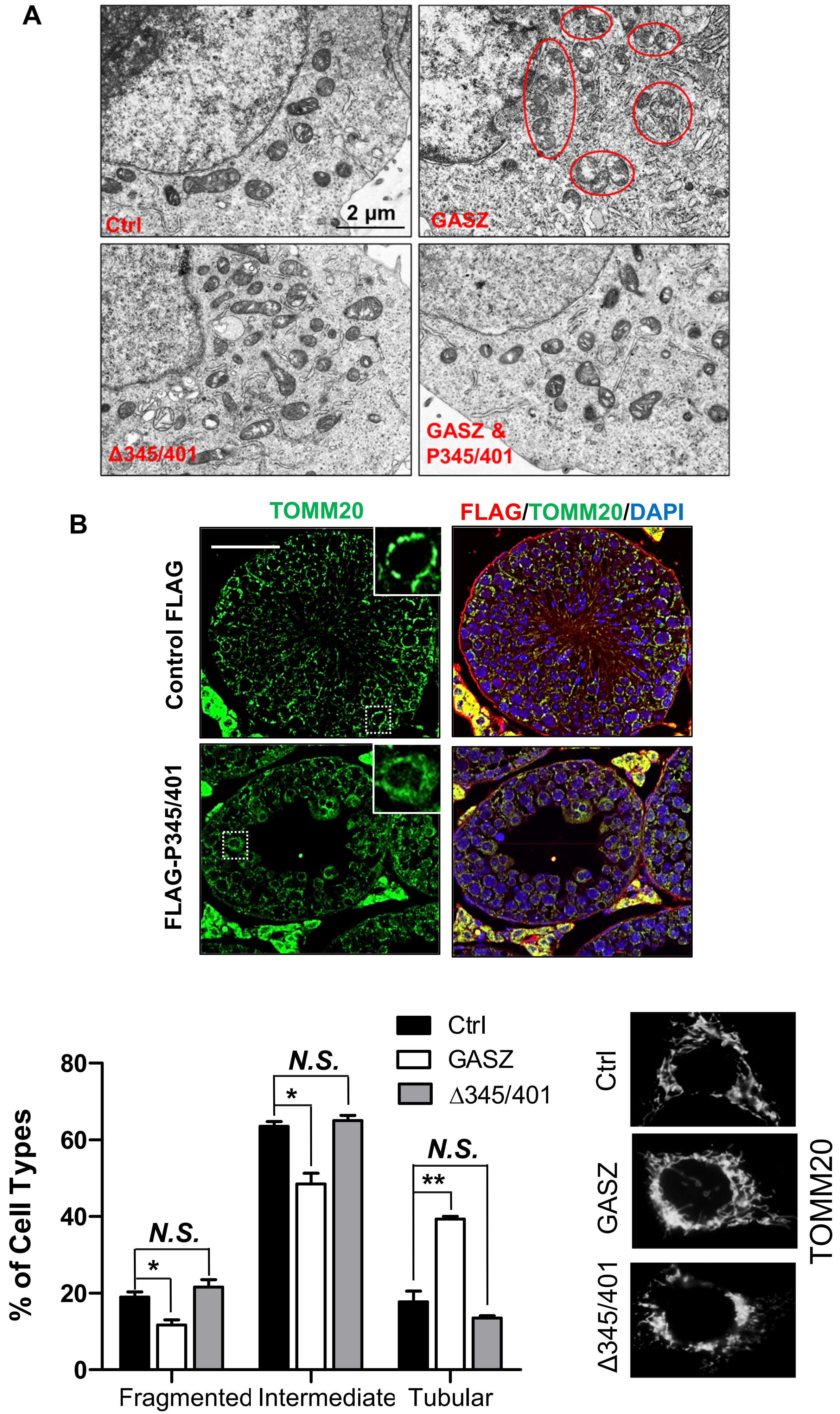

Miao et al., Supplemental Fig. S4

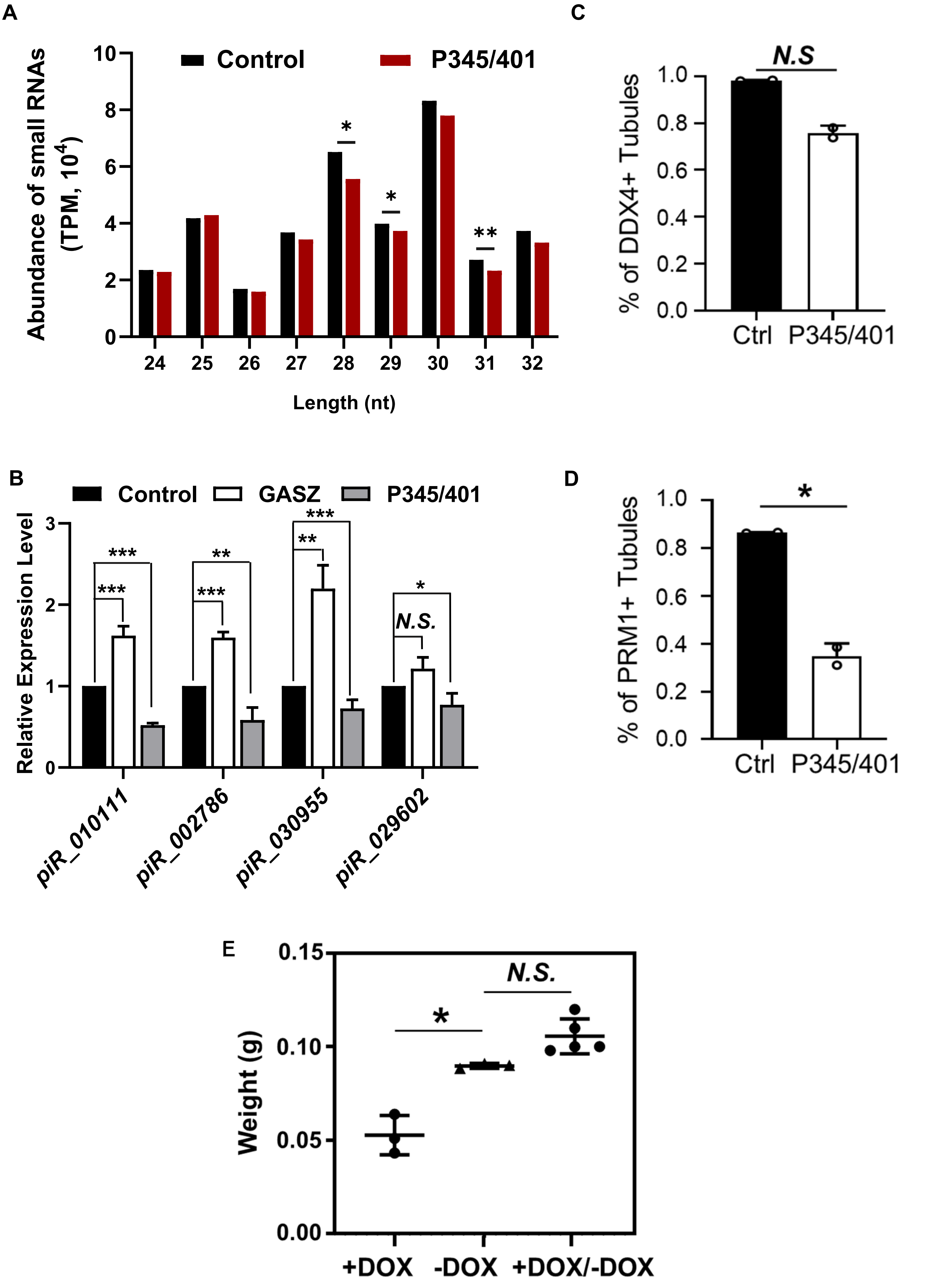

Miao et al., Supplemental Fig. S5
